# Our Associate and Corresponding Editors

**Published:** 1871-09

**Authors:** 


### PART III.

### Editorials, Correspondence and Miscellaneous.

### OUR ASSOCIATE AND CORRESPONDING EDITORS.

Since our last issue we have added many "bright stars" to our "galaxy of names" as Associate and Corresponding Editors of The Companion. We welcome the first named as co-laborers in the fertile field of Southern Medical Literature—too sadly neglected by her sons, in the past. We trust we see the dawning of a "new era," when the glory of Southern talent shall, in harmonious light, blend with that now streaming from other lands—for the illumination of science and the aleviation of human suffering. To our Corresponding Editors we extend "a fraternal hand and heart." In behalf of our Southern brethren we thank them for that Professional courtesy and kindness which so promptly induced them to encourage our medical literature and to aid us in establishing a Medical Journal as an organ of the Profession South, by which its talent may be represented, and its honor and dignity upheld.

By our efforts to found a journal commensurate with the wants of the Profession of our Section, it is not our desire to lessen thereby, the influence and circulation of other medical journals. We would rather enlarge both. We are not of those who believe there are already too many medical journals in the country—we have never seen one yet that was not worth the subscription price.

We thank those who have forwarded us contributions. They will appear in due time. We will still give special attention to our Second Part, in gleaning the best current medical literature coming to us in our exchanges. We ask our friends, everywhere, to send us valuable points in practice, therapeutics, formulæ, etc., for this department, with name attached.

Our editorial space shall be open for the vindication of truth. Truth cannot be circumscribed to special localities. All Professional gentlemen have an interest in the "local" prosperity of his calling, and should feel a "local" insult to its honor and glory a "National" wound. We pity the "head and heart" that can calmly and complacently behold a struggle for professional honor, unmoved—and thus play the role of a neutral, because said "head and heart" has no manly blood or professional sympathy to fire and warm a dastard soul. It is true, THE COMPANION is published on Southern soil, for the development of Southern intellect, and for the promotion of Southern medicine; yet it will defend the right and rebuke the wrong whenever a brother's cause shall ask it. In the Great Medical Brotherhood we recognize no geographical lines. Truth, principle, law, these are our talismen; and wherever found are an implantation of Deity, the insignia of honor, and the bonds of a common faith and a common heritage.

